# Supplementary material for: Diagnostic Accuracy of Machine Learning Models on Mammography in Breast Cancer Classification: A Meta-Analysis
Source: Diagnostics (Basel). 2022 Jul 5;12(7):1643. doi: 10.3390/diagnostics12071643 (PMC9320089; doi:10.3390/diagnostics12071643)
Supplement: Supplementary file 1 [file diagnostics-12-01643-s001.zip › Supp File.pdf]

PRISMA-DTA Checklist

Section/topic	#	PRISMA-DTA Checklist Item	Reported on page #
**TITLE / ABSTRACT**			
Title	1	Identify the report as a systematic review (+/- meta-analysis) of diagnostic test accuracy (DTA) studies.	1
Abstract	2	Abstract: See PRISMA-DTA for abstracts.	1
**INTRODUCTION**			
Rationale	3	Describe the rationale for the review in the context of what is already known.	1-2
Clinical role of index test	D1	State the scientific and clinical background, including the intended use and clinical role of the index test, and if applicable, the rationale for minimally acceptable test accuracy (or minimum difference in accuracy for comparative design).	2
Objectives	4	Provide an explicit statement of question(s) being addressed in terms of participants, index test(s), and target condition(s).	2
**METHODS**			
Protocol and registration	5	Indicate if a review protocol exists, if and where it can be accessed (e.g., Web address), and, if available, provide registration information including registration number.	NA
Eligibility criteria	6	Specify study characteristics (participants, setting, index test(s), reference standard(s), target condition(s), and study design) and report characteristics (e.g., years considered, language, publication status) used as criteria for eligibility, giving rationale.	2
Information sources	7	Describe all information sources (e.g., databases with dates of coverage, contact with study authors to identify additional studies) in the search and date last searched.	2
Search	8	Present full search strategies for all electronic databases and other sources searched, including any limits used, such that they could be repeated.	2
Study selection	9	State the process for selecting studies (i.e., screening, eligibility, included in systematic review, and, if applicable, included in the meta-analysis).	2
Data collection process	10	Describe method of data extraction from reports (e.g., piloted forms, independently, in duplicate) and any processes for obtaining and confirming data from investigators.	3
Definitions for data extraction	11	Provide definitions used in data extraction and classifications of target condition(s), index test(s), reference standard(s) and other characteristics (e.g. study design, clinical setting).	NA
Risk of bias and applicability	12	Describe methods used for assessing risk of bias in individual studies and concerns regarding the applicability to the review question.	3
Diagnostic accuracy measures	13	State the principal diagnostic accuracy measure(s) reported (e.g. sensitivity, specificity) and state the unit of assessment (e.g. per-patient, per-lesion).	3
Synthesis of results	14	Describe methods of handling data, combining results of studies and describing variability between studies. This could include, but is not limited to: a) handling of multiple definitions of target condition. b) handling of multiple thresholds of test positivity, c) handling multiple index test readers, d) handling of indeterminate test results, e) grouping and comparing tests, f) handling of different reference standards	3-4

PRISMA-DTA Checklist

Section/topic	#	PRISMA-DTA Checklist Item	Reported on page #
Meta-analysis	D2	Report the statistical methods used for meta-analyses, if performed.	3-4
Additional analyses	16	Describe methods of additional analyses (e.g., sensitivity or subgroup analyses, meta-regression), if done, indicating which were pre-specified.	3-4
**RESULTS**			
Study selection	17	Provide numbers of studies screened, assessed for eligibility, included in the review (and included in meta-analysis, if applicable) with reasons for exclusions at each stage, ideally with a flow diagram.	4-5
Study characteristics	18	For each included study provide citations and present key characteristics including: a) participant characteristics (presentation, prior testing), b) clinical setting, c) study design, d) target condition definition, e) index test, f) reference standard, g) sample size, h) funding sources	5-8
Risk of bias and applicability	19	Present evaluation of risk of bias and concerns regarding applicability for each study.	14-15
Results of individual studies	20	For each analysis in each study (e.g. unique combination of index test, reference standard, and positivity threshold) report 2x2 data (TP, FP, FN, TN) with estimates of diagnostic accuracy and confidence intervals, ideally with a forest or receiver operator characteristic (ROC) plot.	6-11
Synthesis of results	21	Describe test accuracy, including variability; if meta-analysis was done, include results and confidence intervals.	9-11
Additional analysis	23	Give results of additional analyses, if done (e.g., sensitivity or subgroup analyses, meta-regression; analysis of index test: failure rates, proportion of inconclusive results, adverse events).	11-14
**DISCUSSION**			
Summary of evidence	24	Summarize the main findings including the strength of evidence.	15-16
Limitations	25	Discuss limitations from included studies (e.g. risk of bias and concerns regarding applicability) and from the review process (e.g. incomplete retrieval of identified research).	16
Conclusions	26	Provide a general interpretation of the results in the context of other evidence. Discuss implications for future research and clinical practice (e.g. the intended use and clinical role of the index test).	15-16
**FUNDING**			
Funding	27	For the systematic review, describe the sources of funding and other support and the role of the funders.	17

Adapted From: McInnes MDF, Moher D, Thombs BD, McGrath TA, Bossuyt PM, The PRISMA-DTA Group (2018). Preferred Reporting Items for a Systematic Review and Meta-analysis of Diagnostic Test Accuracy Studies: The PRISMA-DTA Statement. JAMA. 2018 Jan 23;319(4):388-396. doi: 10.1001/jama.2017.19163.

For more information, visit: **www.prisma-statement.org**.

APPENDIX

SEDATE (UPDATE) Checklist on reporting a diagnostic test accuracy meta-analysis

Section	#	*Item*	*Page*
**TITLE**			
Title	1	Identify report as a systematic review with or without meta-analysis	1
**ABSTRACT**			
Introduction	2	Current knowledge gap this study fills, objective	1
Methods	3	Search strategy, eligibility criteria, outcomes and statistical analysis	1
Results	4	Study selection, main results (numerical estimates and 95% confidence intervals)	1
Conclusion	5	Clinical interpretation of results, limitations and suggestions for future research (if applicable)	1
**INTRODUCTION**			
Rationale	6	Background, information about disease burden, current tests and reference test (if applicable)	1-2
Objective	7	Clearly describe primary and secondary (if applicable) aims of the study	2
**METHODS**			
Protocol and registration	8	Indicate if a study protocol was drafted before performing literature search and extraction of data, indicate if protocol was registered with a database (e.g. PROSPERO) and, if so, provide identification number	NA
Eligibility criteria	9	Provide details about: (1) types of studies, (2) participants, (3) index test, (4) reference test, (5) outcome, (6) setting, (7) data required for extraction, (8) study size and language	2-3
Information sources	10	Report details of search strategy: databases, dates, terms, additional searches	2
Study selection	11	Describe process of study selection: screening → eligibility → inclusion in systematic review/meta-analysis	2
Risk of bias in individual studies	12	Describe methods used for assessing risk of bias, QUADAS-2 in case of DTA meta-analysis, and provide a short description of items assessed	3
Data items	13	List items retrieved from every study, including, but not confined to, PICOS	3
Summary outcomes and synthesis of results	14	Report pooled outcome measures (e.g. sensitivity, specificity, likelihood ratios, SROC curves), describe methods used for synthesis of results (random-effects models in case of DTA meta-analysis) and analysis of heterogeneity (I^2 and/or SROC curve).	3-4
Additional analyses	15	Report any planned secondary analyses (e.g. sensitivity analysis, subgroup analysis, meta-regression)	3-4
Risk of bias across studies	16	Describe analysis performed for assessing publication bias (e.g. Deeks' test), if applicable.	4
**RESULTS**			
Study selection	17	Report number of studies screened, articles retrieved in full text and articles included in meta-analysis, with numbers and reasons for exclusion between each of these steps	4-5
Flow diagram	18	Provide details of item #17 as a flow diagram	5
Study characteristics and results	19	Provide relevant details of all included studies (e.g. author, country, period of enrollment, eligibility criteria, number of participants), preferably as a table	5-8
Risk of bias within studies	20	Provide results of assessment described in item #12 in the form of a table or figure, and give brief summary in text body	14-15
Synthesis of results	21	Present results of pooled estimates in main text, including number of studies (with references), number of events and 95% confidence intervals and heterogeneity measures for each estimate; repeat for each outcome, if applicable	9-11
	22	Provide numerical results for each included study and pooled estimates (including heterogeneity measures) as a table	11, Supp
	23	In case of binary results provide forest plots for sensitivity and specificity; in case of continuous measures construct hierarchical SROC curves	9-11
Additional analyses	24	Present results of any additional prespecified analysis (e.g. subgroup or sensitivity analyses) as described in item #21; figures for these analyses can be constructed as per item #23	11-13
Publication bias	25	Report results of assessment described in item #15	13-14
**DISCUSSION**			
Summary of evidence	26	Describe main findings, including their numerical estimates	15
Interpretation of the results	27	Discuss the results in their clinical context (e.g. pathophysiological basis, clinical impact, applicability, generalizability)	15-16
Limitations	28	Discuss facts potentially limiting strength of the results, including (but not limited to) paucity of data, methodological limitations of primary studies, bias at meta-analysis level and heterogeneity	16
Conclusions	29	Provide a clinical take-home message of results, highlight their limitations and propose topics for future research, if applicable	16

Copyright © 2015 ISUOG. Published by John Wiley & Sons Ltd.
