# Supplementary material for: Diagnostic Accuracy of Machine Learning Models on Mammography in Breast Cancer Classification: A Meta-Analysis
Source: Diagnostics (Basel). 2022 Jul 5;12(7):1643. doi: 10.3390/diagnostics12071643 (PMC9320089; doi:10.3390/diagnostics12071643)
Supplement: Supplementary file 1 [file diagnostics-12-01643-s001.zip › Table S1- influential-diagnostic.pdf]

Table S1. Result of influential diagnostic of overall machine learning models.

Model	AUC	ΔAUC	Bootstrap 2.5th percentile	Bootstrap 97.5th percentile
All model	0.898			
Excluding model 12	0.894	-0.004	-0.006	-0.006
Excluding model 4	0.895	-0.003	-0.004	-0.004
Excluding model 38	0.902	0.003	-0.004	-0.004
Excluding model 10	0.895	-0.003	-0.005	-0.005
Excluding model 50	0.902	0.003	-0.005	-0.005
Excluding model 52	0.901	0.003	-0.005	-0.005
Excluding model 25	0.896	-0.003	-0.004	-0.004
Excluding model 49	0.901	0.003	-0.005	-0.005
Excluding model 22	0.896	-0.003	-0.004	-0.004
Excluding model 39	0.901	0.003	-0.003	-0.003
Excluding model 40	0.901	0.003	-0.004	-0.004
Excluding model 33	0.901	0.003	-0.004	-0.004
Excluding model 46	0.896	-0.002	-0.006	-0.006
Excluding model 59	0.896	-0.002	-0.003	-0.003
Excluding model 32	0.901	0.002	-0.004	-0.004
Excluding model 3	0.896	-0.002	-0.003	-0.003
Excluding model 62	0.901	0.002	-0.002	-0.002
Excluding model 58	0.896	-0.002	-0.003	-0.003
Excluding model 6	0.896	-0.002	-0.004	-0.004
Excluding model 48	0.9	0.002	-0.004	-0.004
Excluding model 21	0.9	0.002	-0.003	-0.003
Excluding model 36	0.897	-0.002	-0.004	-0.004
Excluding model 37	0.9	0.002	-0.003	-0.003
Excluding model 24	0.897	-0.002	-0.005	-0.005
Excluding model 28	0.897	-0.002	-0.003	-0.003
Excluding model 45	0.9	0.002	-0.002	-0.002
Excluding model 26	0.897	-0.002	-0.004	-0.004
Excluding model 35	0.9	0.002	-0.004	-0.004
Excluding model 34	0.9	0.002	-0.004	-0.004
Excluding model 63	0.9	0.002	-0.002	-0.002
Excluding model 44	0.9	0.002	-0.002	-0.002
Excluding model 30	0.897	-0.002	-0.003	-0.003
Excluding model 66	0.897	-0.002	-0.004	-0.004
Excluding model 11	0.897	-0.001	-0.005	-0.005
Excluding model 18	0.9	0.001	-0.004	-0.004
Excluding model 54	0.897	-0.001	-0.002	-0.002
Excluding model 2	0.897	-0.001	-0.003	-0.003
Excluding model 8	0.9	0.001	-0.004	-0.004
Excluding model 17	0.9	0.001	-0.003	-0.003
Excluding model 55	0.897	-0.001	-0.002	-0.002
Excluding model 47	0.897	-0.001	-0.005	-0.005
Excluding model 1	0.9	0.001	-0.002	-0.002
Excluding model 20	0.9	0.001	-0.003	-0.003
Excluding model 53	0.9	0.001	-0.005	-0.005
Excluding model 64	0.897	-0.001	-0.002	-0.002
Excluding model 7	0.899	0.001	-0.004	-0.004

Excluding model 65	0.898	-0.001	-0.002	-0.002
Excluding model 23	0.898	-0.001	-0.002	-0.002
Excluding model 19	0.899	0.001	-0.004	-0.004
Excluding model 9	0.898	-0.001	-0.002	-0.002
Excluding model 31	0.898	-0.001	-0.004	-0.004
Excluding model 67	0.898	-0.001	-0.002	-0.002
Excluding model 42	0.899	0.001	-0.002	-0.002
Excluding model 27	0.899	0.001	-0.003	-0.003
Excluding model 13	0.899	0	-0.006	-0.006
Excluding model 15	0.898	0	-0.002	-0.002
Excluding model 43	0.898	0	-0.002	-0.002
Excluding model 57	0.898	0	-0.002	-0.002
Excluding model 14	0.899	0	-0.005	-0.005
Excluding model 16	0.898	0	-0.003	-0.003
Excluding model 29	0.899	0	-0.003	-0.003
Excluding model 56	0.898	0	-0.002	-0.002
Excluding model 60	0.898	0	-0.002	-0.002
Excluding model 61	0.899	0	-0.002	-0.002
Excluding model 41	0.898	0	-0.005	-0.005
Excluding model 68	0.899	0	-0.002	-0.002
Excluding model 5	0.899	0	-0.002	-0.002
Excluding model 51	0.899	0	-0.005	-0.005
