# Supplementary material for: Diagnostic Accuracy of Machine Learning Models on Mammography in Breast Cancer Classification: A Meta-Analysis
Source: Diagnostics (Basel). 2022 Jul 5;12(7):1643. doi: 10.3390/diagnostics12071643 (PMC9320089; doi:10.3390/diagnostics12071643)
Supplement: Supplementary file 1 [file diagnostics-12-01643-s001.zip › Table S2 - Search terms.pdf]

Table S2. Search terms used in the study.

Databases	Search terms
PubMed	(("breast cancer"[Title] OR "breast cancers"[Title] OR "breast neoplasm"[Title] OR "breast neoplasms"[Title] OR "breast carcinoma"[Title] OR "breast carcinomas"[Title] OR "ductal carcinoma"[Title] OR "ductal carcinomas"[Title] OR "breast mass"[Title] OR "breast lesion"[Title] OR "breast lesions"[Title] OR "breast tumour"[Title] OR "breast tumours"[Title] OR "breast tumor"[Title] OR "breast tumors"[Title] OR "breast malignancy"[Title] OR "breast malignancies"[Title] OR "mammary cancer"[Title] OR "mammary cancers"[Title]) AND (Mammograph[Title/Abstract] OR Mammographs[Title/Abstract] OR Mammographic[Title/Abstract] OR Mammography[Title/Abstract] OR Mammographies[Title/Abstract] OR Mammogram[Title/Abstract] OR Mammograms[Title/Abstract] OR Tomosynthesis[Title/Abstract] OR Tomosyntheses[Title/Abstract])) AND ("machine learning"[Title/Abstract] OR "machine learnings"[Title/Abstract] OR "deep learning"[Title/Abstract] OR "Decision tree"[Title/Abstract] OR "Classification tree"[Title/Abstract] OR "Tree-based"[Title/Abstract] OR "Support vector"[Title/Abstract] OR "Transfer learning"[Title/Abstract] OR "neural network"[Title/Abstract] OR "neural networks"[Title/Abstract] OR "Logistic regression"[Title/Abstract] OR "discriminant analysis"[Title/Abstract] OR "k-nearest neighbor"[Title/Abstract] OR "k-nearest neighbour"[Title/Abstract] OR "nearest neighbor"[Title/Abstract] OR "nearest neighbour"[Title/Abstract] OR bayes[Title/Abstract] OR "random forest"[Title/Abstract])
Scopus	TITLE("breast cancer" OR "breast cancers" OR "breast neoplasm" OR "breast neoplasms" OR "breast carcinoma" OR "breast carcinomas" OR "ductal carcinoma" OR "ductal carcinomas" OR "breast mass" OR "breast lesion" OR "breast lesions" OR "breast tumour" OR "breast tumours" OR "breast tumor" OR "breast tumors" OR "breast malignancy" OR "breast malignancies" OR "mammary cancer" OR "mammary cancers") AND TITLE-ABS(Mammograph OR Mammographs OR Mammographic OR Mammography OR Mammographies OR Mammogram OR Mammograms OR Tomosynthesis OR Tomosyntheses) AND TITLE-ABS("machine learning" OR "machine learnings" OR "deep learning" OR "Decision tree" OR "Classification tree" OR "Tree-based" OR "Support vector" OR "Transfer learning" OR "neural network" OR "neural networks" OR "Logistic regression" OR "discriminant analysis" OR "k-nearest neighbor" OR "k-nearest neighbour" OR "nearest neighbor" OR "nearest neighbour" OR bayes OR "random forest")
Google Scholar	1- (Mammograph OR Mammographs OR Mammographic OR Mammography OR Mammographies OR Mammogram OR Mammograms OR Tomosynthesis OR Tomosyntheses) (breast) ("machine learning")

	2- (Mammograph OR Mammographs OR Mammographic OR Mammography OR Mammographies OR Mammogram OR Mammograms OR Tomosynthesis OR Tomosyntheses) (breast) ("machine learnings")<br>3- (Mammograph OR Mammographs OR Mammographic OR Mammography OR Mammographies OR Mammogram OR Mammograms OR Tomosynthesis OR Tomosyntheses) (breast) ("deep learning")<br>4- (Mammograph OR Mammographs OR Mammographic OR Mammography OR Mammographies OR Mammogram OR Mammograms OR Tomosynthesis OR Tomosyntheses) (breast) ("Decision tree")<br>5- (Mammograph OR Mammographs OR Mammographic OR Mammography OR Mammographies OR Mammogram OR Mammograms OR Tomosynthesis OR Tomosyntheses) (breast) ("Classification tree”)<br>6- (Mammograph OR Mammographs OR Mammographic OR Mammography OR Mammographies OR Mammogram OR Mammograms OR Tomosynthesis OR Tomosyntheses) (breast) ("Tree-based")<br>7- (Mammograph OR Mammographs OR Mammographic OR Mammography OR Mammographies OR Mammogram OR Mammograms OR Tomosynthesis OR Tomosyntheses) (breast) ("Support vector")<br>8- (Mammograph OR Mammographs OR Mammographic OR Mammography OR Mammographies OR Mammogram OR Mammograms OR Tomosynthesis OR Tomosyntheses) (breast) ("Transfer learning")<br>9- (Mammograph OR Mammographs OR Mammographic OR Mammography OR Mammographies OR Mammogram OR Mammograms OR Tomosynthesis OR Tomosyntheses) (breast) ("neural network")<br>10- (Mammograph OR Mammographs OR Mammographic OR Mammography OR Mammographies OR Mammogram OR Mammograms OR Tomosynthesis OR Tomosyntheses) (breast) ("neural networks")<br>11- (Mammograph OR Mammographs OR Mammographic OR Mammography OR Mammographies OR Mammogram OR Mammograms OR Tomosynthesis OR Tomosyntheses) (breast) ("Logistic regression")<br>12- (Mammograph OR Mammographs OR Mammographic OR Mammography OR Mammographies OR Mammogram OR Mammograms OR Tomosynthesis OR Tomosyntheses) (breast) ("discriminant analysis")<br>13- (Mammograph OR Mammographs OR Mammographic OR Mammography OR Mammographies OR Mammogram OR Mammograms OR Tomosynthesis OR Tomosyntheses) (breast) ("k-nearest neighbor")<br>14- (Mammograph OR Mammographs OR Mammographic OR Mammography OR Mammographies OR Mammogram OR

	Mammograms OR Tomosynthesis OR Tomosyntheses) (breast) ("k-nearest neighbour")<br>15- (Mammograph OR Mammographs OR Mammographic OR Mammography OR Mammographies OR Mammogram OR Mammograms OR Tomosynthesis OR Tomosyntheses) (breast) ("nearest neighbor")<br>16- (Mammograph OR Mammographs OR Mammographic OR Mammography OR Mammographies OR Mammogram OR Mammograms OR Tomosynthesis OR Tomosyntheses) (breast) ("bayes")<br>17- (Mammograph OR Mammographs OR Mammographic OR Mammography OR Mammographies OR Mammogram OR Mammograms OR Tomosynthesis OR Tomosyntheses) (breast) ("random forest")
Web of Science	TI=("breast cancer" OR "breast cancers" OR "breast neoplasm" OR "breast neoplasms" OR "breast carcinoma" OR "breast carcinomas" OR "ductal carcinoma" OR "ductal carcinomas" OR "breast mass" OR "breast lesion" OR "breast lesions" OR "breast tumour" OR "breast tumours" OR "breast tumor" OR "breast tumors" OR "breast malignancy" OR "breast malignancies" OR "mammary cancer" OR "mammary cancers") AND TI=(Mammograph OR Mammographs OR Mammographic OR Mammography OR Mammographies OR Mammogram OR Mammograms OR Tomosynthesis OR Tomosyntheses) AND TI=("machine learning" OR "machine learnings" OR "deep learning" OR "Decision tree" OR "Classification tree" OR "Tree-based" OR "Support vector" OR "Transfer learning" OR "neural network" OR "neural networks" OR "Logistic regression" OR "discriminant analysis" OR "k-nearest neighbor" OR "k-nearest neighbour" OR "nearest neighbor" OR "nearest neighbour" OR bayes OR "random forest")
Embase	(‘breast cancer’:ti OR ‘breast cancers’:ti OR ‘breast neoplasm’:ti OR ‘breast neoplasms’:ti OR ‘breast carcinoma’:ti OR ‘breast carcinomas’:ti OR ‘ductal carcinoma’:ti OR ‘ductal carcinomas’:ti OR ‘breast mass’:ti OR ‘breast lesion’:ti OR ‘breast lesions’:ti OR ‘breast tumour’:ti OR ‘breast tumours’:ti OR ‘breast tumor’:ti OR ‘breast tumors’:ti OR ‘breast malignancy’:ti OR ‘breast malignancies’:ti OR ‘mammary cancer’:ti OR ‘mammary cancers’:ti) AND (Mammograph:ti OR Mammographs:ti OR Mammographic:ti OR Mammography:ti OR Mammographies:ti OR Mammogram:ti OR Mammograms:ti OR Tomosynthesis:ti OR Tomosyntheses:ti) AND (‘machine learning’:ti OR ‘machine learnings’:ti OR ‘deep learning’:ti OR ‘Decision tree’:ti OR ‘Classification tree’:ti OR ‘Tree-based’:ti OR ‘Support vector’:ti OR ‘Transfer learning’:ti OR ‘neural network’:ti OR ‘neural networks’:ti OR ‘Logistic regression’:ti OR ‘discriminant analysis’:ti OR ‘k-nearest neighbor’:ti OR ‘k-nearest neighbour’:ti OR ‘nearest neighbor’:ti OR ‘nearest neighbour’:ti OR bayes:ti OR ‘random forest’:ti)
